# Current Updates on Variants of SARS‐CoV‐ 2: Systematic Review

**DOI:** 10.1002/hsr2.70166

**Published:** 2024-11-04

**Authors:** Mulat Erkihun, Bayu Ayele, Zelalem Asmare, Kirubel Endalamaw

**Affiliations:** ^1^ Department of Medical Laboratory Sciences, School of Health Sciences, College of Medicine and Health Sciences Debre Tabor University Debre Tabor Ethiopia; ^2^ Laboratory Service Unit Felege Hiwot Comprehensive Specialized Hospital Bahir Dar Ethiopia; ^3^ Department of Medical Laboratory Sciences, College of Health Sciences Woldia University Woldia Ethiopia; ^4^ Department of Diagnostic Laboratory at Shegaw Motta General Hospital Motta Town Ethiopia

**Keywords:** COVID‐19, SARS‐CoV‐2, variants

## Abstract

**Background:**

Coronavirus disease 2019 is caused by the severe acute respiratory syndrome coronavirus 2, which has become a pandemic. Severe acute respiratory syndrome coronavirus 2 is an enveloped, unsegmented, positive‐sense, single‐stranded RNA virus that belongs to the family Coronaviridae.

**Aim:**

The objective of this review is to conduct a qualitative analysis of the current updates on epidemiology, evolution, and vaccine variants for SARS‐CoV‐2.

**Method:**

The search strategy was done from the database based on the PRISMA criteria for qualitative analysis of this review. Literature on variants of severe acute respiratory syndrome coronavirus 2, published in English in the last 5 years (2019–2023), were included. From 179 a total of 105 articles were reviewed, searched, and retrieved from the electronic databases PubMed. The search was done using keywords like COVID‐19, SARS‐CoV‐2, variants, mutations, and vaccines, and articles were managed using EndNote X8 software. The scope of view for this review was the course of the pandemic by emerging variants and how man is struggling to overcome this sudden pandemic through vaccines. The narrative skeleton was constructed based on the article's scope of view.

**Result:**

From the parent severe acute respiratory syndrome coronavirus 2, many variants emerged during the course of this pandemic. They are mainly categorized into two variants: variants of interest and variants of concern based on the impact on public health. The World Health Organization leveled five variants: Alpha (strain B.1.1.7), Beta (strain B.1.351), Gamma (strain P.1), Delta (strain B.1.617.2), and Omicron (B.1.1.529).

**Conclusions:**

It is crucial to stay informed about the latest developments in the understanding of SARS‐CoV‐2 variants, as new variants can emerge and impact the course of the pandemic. Health authorities and researchers continuously have to monitor and study these variants to assess their characteristics, transmissibility, severity, and the effectiveness of vaccines against them. One has to always refer to the latest information from reputable health journals or organizations for the most up‐to‐date and accurate details on COVID‐19 variants.

**Abbreviations**
ACE2: angiotensin‐converting enzyme 2
APC: antigen presenting cells
ARDS: acute respiratory distress syndrome
COVID‐19: Coronavirus Disease‐19
DNA: deoxyribonucleic acid
HIV: human immuno virus
LRT: lower respiratory tract
MERS‐CoV: middle east respiratory syndrome coronavirus
mRNA: messenger ribonucleic acid
NSP: nonstructural protein
NTD: N‐terminal domain,
ORF: open reading frame
RBD: receptor‐binding domain
RBM: receptor binding motif
RNA: ribonucleic acid
RTC: replication transcriptions complex
SARS‐CoV: severe acute respiratory syndrome coronavirus
SARS‐CoV‐2: severe acute respiratory syndrome coronavirus ‐2
TMPRSS2: trans‐membrane protease serine 2
URT: upper respiratory tract
VOC: variant of concern
VOI: variant of interest
WHO: World Health Organization

## 1 Introduction

Coronavirus Disease 2019 (COVID‐19) is a current global public health problem with significant mortality and morbidity worldwide. Since its outbreak in Hubei Province, China, in late 2019 (December 2019), COVID‐19 has spread to several countries around the world and is now a pandemic [1, 2]. This outbreak had spread to all over the world May 24, 2022, with more than 528,387,692 cases [139,498,808] recovered and cumulative deaths of 6,301,922 worldwide [3]. In Ethiopia, from January 3, 2020, to October 6, 2022, there have been 493,588 confirmed cases of COVID‐19, with 7572 deaths, reported to the World Health Organization (WHO) [4].

The virus that causes COVID‐19 is severe acute respiratory syndrome coronavirus 2 (SARS‐CoV‐2). It is an unsegmented, positive‐sense, enveloped, single‐stranded RNA virus that is a member of the family Coronaviridae, order Nidovirals, and genus Coronaviruses. It is one of the most complex viruses, having a 3′poly‐A tail and a 5′‐cap structure, and a genome size of 26–32 kilobases [5, 6].

Similar to other RNA viruses, SARS‐CoV‐2 has a tendency to change throughout transmissible propagation, giving rise to a variety of new variations. The COVID‐19 pandemic has seen a rapid spread of SARS‐CoV‐2 due to its tolerance to a variety of cellular settings and varied hosts. Meanwhile, SARS‐CoV‐2 spreads swiftly and exacerbates symptoms due to genetic sequence mutations [7]. Genetic sequence alterations, meantime, contribute to the rapid spread and aggravation of SARS‐CoV‐2 [7, 8].

SARS‐CoV‐2 has 29 genes and its with 29,900 nucleotides. It codes 16 nonstructural proteins (NSPsand four structural proteins: spike (S), envelope (E), membrane (M), and nucleocapsid (N). Viral glycoprotein S binds to the angiotensin‐converting enzyme 2 (ACE2) receptor, which is the same receptor that SARS‐CoV uses to enter human cells. The S protein is made up of two subunits: the S1 subunit, which is responsible for containing the receptor‐binding domain (RBD), and the S2 subunit, which facilitates the fusing of the virus with the host cell membrane following its primary trimming by trans‐membrane serine protease 2 (TMPRSS2). Although there are other sections of spike (S protein) that also trigger neutralizing activity (NAc), the RBD region is the principal focus of cytotoxic lymphocytes and neutralizing antibodies (NAbs) [9].

Numerous SARS‐CoV‐2 variations have been identified and documented subsequent to the onset of the COVID‐19 epidemic. New mutations or variants may arise due to a variety of causes, including natural selection, immune‐compromised individuals' recurrent infections, random events, host shifts, mutations involving a lack of proofreading ability, the co‐infection mediated hybrid viral formation and mutations during translations [10]. An in vivo selection of mutations resistant to antibody responses may have occurred for some of the developing variant mutations found in immune‐compromised individuals with persistent viral shedding and in spontaneous non‐responders.

The SARS‐CoV‐2 pandemic has resulted in the description of multiple variants. They were divided into two groups. These are known as variants of interest (VOI) and variants of concern (VOC) [11]. The WHO has classified only a small number of these as VOCs due to their potential to negatively impact public health worldwide. A mutated strain that is more transmissible, leads to a more severe disease progression, increases mortality, eludes antibody neutralization, and/or is difficult to detect is known as a VOC. Five SARS‐CoV‐2 VOCs have been identified since the start of the pandemic: Alpha (B.1.1.7), Beta (B.1.351), Gamma (P1), Delta (B.1.617.2), Omicron (B.1.1.529), and all of their sub‐variants are currently referred to as VOC [12, 13].

Genomic sequencing carried out in Ethiopia and at Kenya Medical Research Institute (KEMRI) has identified Alpha, Beta, Delta, and Omicron variants among the SARS‐CoV‐2 VOC [14, 15, 16].

Based on an analysis of the evolutionary relationships between SARS‐CoV‐2 viruses and samples collected before April 2021, it was found that 93% of the viruses belonged to the Alpha, Beta, and Gamma emerging lineages in the ongoing COVID‐19 pandemic. Delta variant frequencies rose quickly, from 1% in April 2021 to 91% in November 2021 [17]. A number of noteworthy SARS‐CoV‐2 variants have surfaced due to the possibility of enhanced transmissibility. At the moment, B.1.1.529 (Omicron) is becoming more frequent. The current challenge to SARS‐CoV‐2 vaccines is the emergence of multiple highly transmissible variants. Thus, the purpose of this review is to present an up‐to‐date overview of the epidemiology and evolutionary history of SARS CoV‐2 variants [14].

## 2 Methods

A literature search was taken from PubMed to identify all published or electronically published English‐language articles relevant to Current Updates on Variants of SARS‐CoV‐2. Out of 179 records, a total of 105 were included in the time frame of 2019–2023. The search was done for clinical trials, systematic reviews, reviews, randomized controls and research articles which could be relevant information sources for this work. In PubMed, the following search string was used: ((((((COVID19) OR (SARS‐ COV‐ 2)) AND (Alpha Variant (B.1.1.7))) AND (Beta variant (B.1.135)) AND (Gamma variant (P.1)) AND (Delta Variant (B.1.617.2)) AND (vaccine)). The exclusion criteria were only abstracts, not English language articles, books, antiviral trials other than vaccine.

### 2.1 Molecular Characteristics of SARS‐CoV‐2 Variants

**Figure 1:**
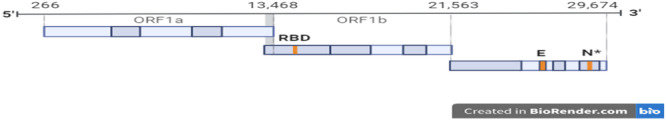
The genome structure of SARS‐CoV‐2 viruses.

With a genome of about 30 kb, SARS‐CoV‐2 is a single‐stranded positive‐sense RNA (+ssRNA) virus that contains 14 open‐reading frames (ORFs) that encode the various viral proteins [6, 18]. Four structural proteins—the spike (S), membrane (M), envelope (E), and nucleo‐capsid (N) proteins—and 16 nonstructural proteins (NSPs) are encoded by these 14 open reading frames (Figure 1). The spike protein splits into S1 and S2 subunits. RBD‐containing S1 is involved in the entry of viruses into host cells [19, 20].

**Figure 2:**
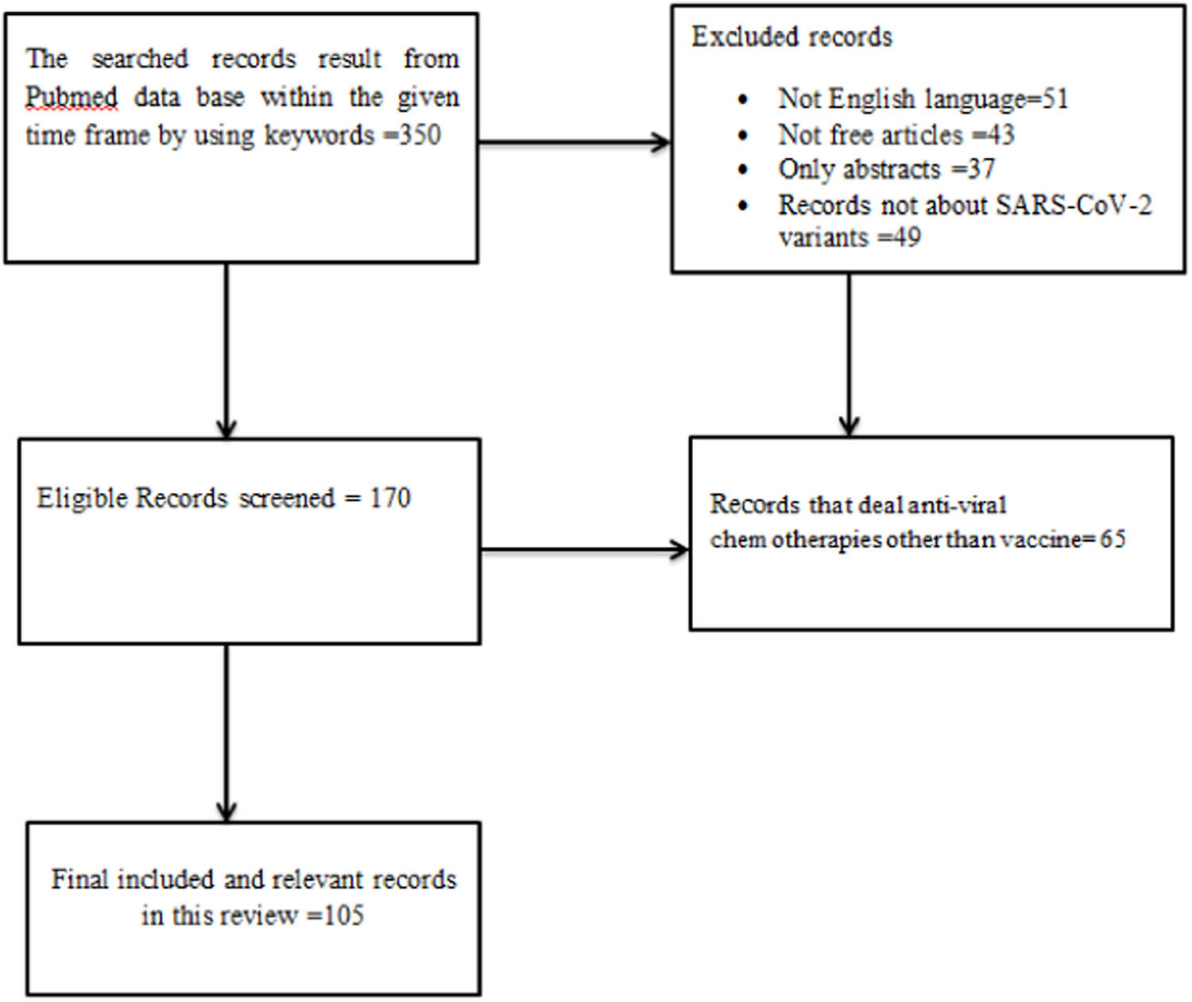
Records selection procedures from database based on the PRISMA criteria for qualitative analysis of this review.

The Wuhan strain's sequences of accessory proteins and those of the NSP and S proteins exhibit distinct substitutions of amino acids, according to a comparison. The virus avoids Nabs, the substitution stabilizes the S protein in human ACE2, and COVID19 becomes more severe [21]. There is greater preservation of membrane (M) and envelope (E) proteins. The E and M proteins' single amino acid substitutions remain unchanged (Figure 2).

SARS‐CoV‐2 has a tendency to change during transmissible progression, giving rise to a variety of novel variants. The COVID‐19 pandemic has seen a rapid spread of SARS‐CoV‐2 due to its adaptability to a variety of cellular environments and diverse hosts. In the meantime, SARS‐CoV‐2 spreads swiftly and exacerbates conditions due to genetic sequence mutations [16]. The S gene in particular has the capacity to mutate into a more infectious form. This mutation has been identified as a clinical VOC and is thought to be the reason for antibodies' increased transmissibility and immune escape. The CDC and the WHO have separately developed a classification system to differentiate the emerging variants of SARS‐CoV‐2 into VOIs and VOCs due to the ongoing emergence of multiple variants [22, 23, 24, 25].

If a strain is known to cause multiple COVID‐19 cases or community transmission, it is assigned a VOI status. They are frequently only occasionally found, and in certain nations, the initial report of them has been linked to a subsequent rise in cases. Mutations in binding receptors that are significant in VOC are present in many of the VOIs. When a variant is found in several countries and either the strain increases the transmissibility of COVID‐19 epidemiology, increases virulence or modifies the clinical presentation of the disease, or reduces the efficacy of social and public health interventions or available diagnostics, the strain is classified as an emergent strain (VOC) (Table 1). Consequently, compared to VOIs, VOCs are more infectious and generally more virulent because they are capable of causing severe diseases and hospitalization [26].

**Table 1:** Summary of Variants of Concern (VOCs) of SARS‐CoV‐2 based on World Health Organization (WHO) (as of August 31, 2021) [27].

WHO name	Geographic region of first detection	Date first detected	Spike mutation of interest	Scientific name (pango lineage)	Characteristics	Reference
Alpha variant	United kingdom	Sep,2020	N501Y,D614G,P681H	B.1.1.7	Rapid transmissibility and higher infectivity	[14, 28]
	Ethiopia	July 13, 2021				
Beta variant	South Africa	Dec,2020	K417N,E484K,N501Y,D614G,A701V	B.1.351	Higher viral infectivity and immune escape	[14, 29]
	Ethiopia	July 13, 2021				
Gamma variant	Brazil	Jan11,2021	K41T,E484K,N501Y,D614G,H655Y	P.1	Augment of viral transmissibility	[30]
Delta variant	India	May11,2021	L452R,T478K,D614G,P681R	B.1.617.2	Most contagious; higher viral replication; and leading to severe illness	[14, 31]
	Ethiopia	Sept. 07, 2021				
Omicron variant	South Africa	November 26, 2021	N969K, Q954H, N440K,D614G	B.1.1.529	Increased viral replication, infectivity and re‐infection; increased transmissibility; immune escape; recombination with HCoV‐229E viruses	[32]

### 2.2 Laboratory Diagnosis

The need for change in the form of more advanced and flexible diagnostic techniques for the identification of SARS‐CoV‐2 infections has undoubtedly been exacerbated by the appearance of new and developing SARS‐CoV‐2 strains. However, because of new variations and a range of symptoms in infected persons, it was more difficult to build swift and sensitive diagnostic tools [8]. The major targets of SARS‐CoV‐2 detection technologies are anti‐SARS‐CoV‐2 antibodies (serological testing), particular viral nucleic acids (molecular testing), and proteins (antigen testing) [33, 34, 35]. The selection of appropriate tests, samples, and timing is crucial in selecting any of these tests because the detection of viral nucleic acids, antigens, and antibodies vary depending on the stage of infection and the variants [19, 36, 37, 38].

### 2.3 Mutations in Emerging SARS‐CoV‐2 Variants

**Figure 3:**
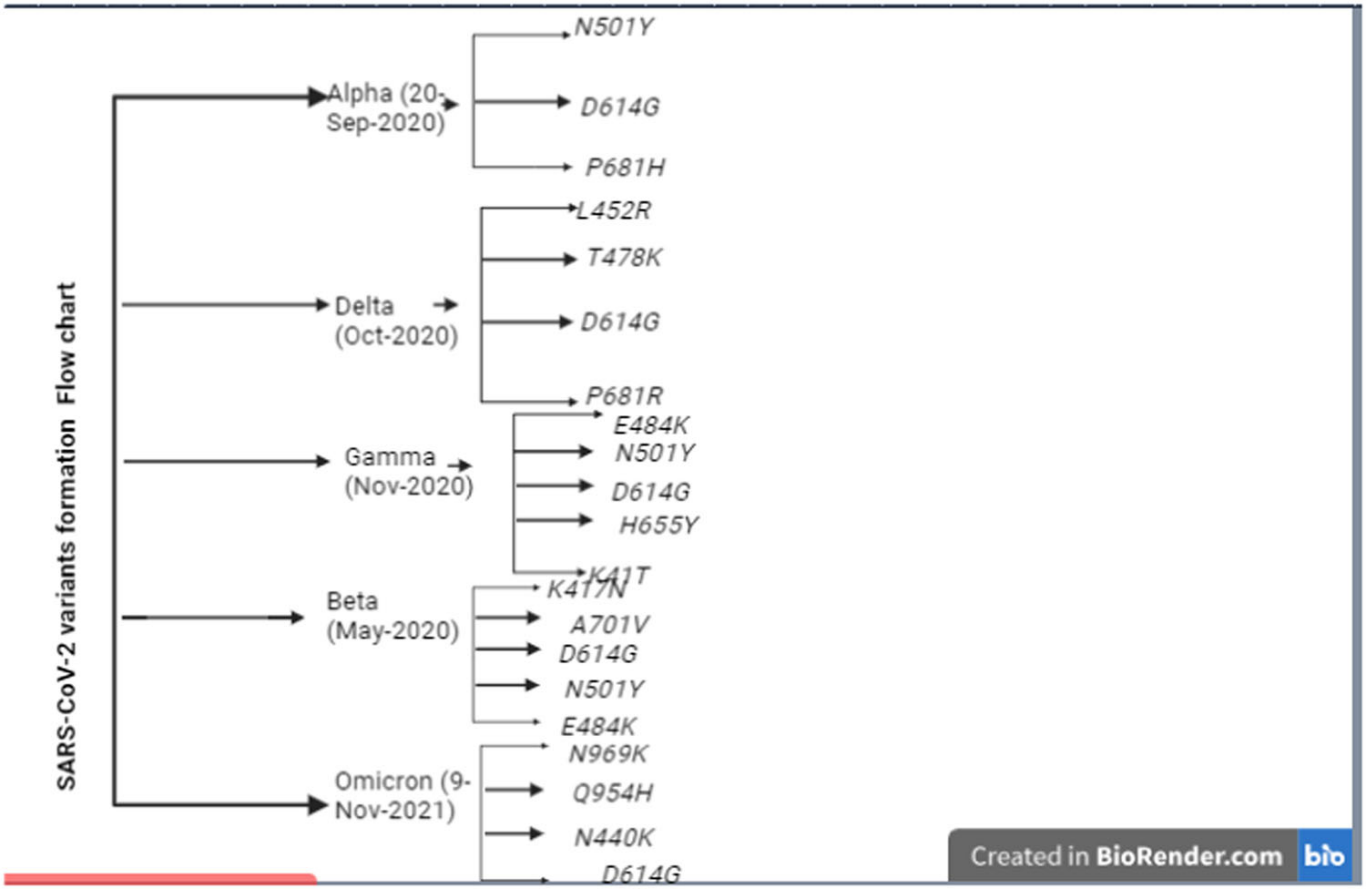
The variants linage and sub‐linage flowcharts within five main variants.

SARS‐CoV‐2 VOCs and VOIs that are currently in circulation share a number of mutations that allow them to persist in the face of increasing population immunity while preserving or enhancing their replication fitness. These mutations are a part of a recurrent mutation repertoire, the majority of which are found in the spike gene. It is vital to comprehend the component mutations of the growing number of SARS‐CoV‐2 VOCs and VOIs to comprehend their biological attributes and epidemiological traits. The RBD mutations N501Y, E484K, D614G, other RBD mutations, NTD mutations, mutations close to the S1/S2 furin cleavage site, and non‐spike mutations are the seven categories of mutations (Figure 3 and Table 1) [38, 39].

### 2.4 D614GG

The D614G mutation is not associated with any particular emerging variant of SARS‐CoV‐2; rather, it is a mutation that became prevalent early in the pandemic. The D614G mutation involves a change in the amino acid at position 614 in the spike (S) protein of the virus. The original form of the virus had an aspartic acid (D) at this position, and the mutation resulted in a substitution with glycine (G) [40].

The D614G mutation has been associated with increased transmissibility of the virus. Studies have suggested that viruses carrying the G614 variant may be more infectious than those with the original D614 variant. However, there is currently no evidence to suggest that the D614G mutation increases the severity of the disease or affects the efficacy of vaccines [41].

It's worth noting that this mutation became dominant globally during the early months of the pandemic, and it is present in most if not all of the major variants that have been identified, including Alpha, Beta, Gamma, Delta, and others (Figure 3).

As the understanding of the virus evolves, it's essential to stay updated with the latest research and information from reputable health organizations for the most current knowledge of SARS‐CoV‐2 and its variants [42].

### 2.5 N501Y

The N501Y mutation is a significant mutation found in the spike protein of SARS‐CoV‐2, the virus responsible for COVID‐19. This mutation involves a change in the amino acid at position 501 of the spike protein, where the original amino acid asparagine (N) is replaced by tyrosine (Y) [43]. The N501Y mutation has been identified in several emerging variants of SARS‐CoV‐2 and has been associated with increased transmissibility. Some of the variants that carry the N501Y mutation include:

#### 2.5.1 Alpha Variant (B.1.1.7)

This variant was first identified in the United Kingdom and has the N501Y mutation, among others.

#### 2.5.2 Beta Variant (B.1.135)

Originated from South Africa and carries N501Y mutation with other mutations.

#### 2.5.3 Gamma Variant (P.1)

Initially originated from in Brazil and it is also with N501Y mutation.

#### 2.5.4 Delta Variant (B.1.617.2)

Delta variant also originated in India and carried N501Y mutation with other key mutations.

The N501Y mutation is located in the RBD of the spike protein, which is involved in binding to the ACE2 receptor on human cells. Changes in this region can potentially affect the virus's ability to enter and infect human cells. The increased transmissibility associated with the N501Y mutation has raised concerns about the potential for more rapid spread of these variants. In general N501Y increases ACE2 affinity and increases virus replication in human upper‐airway cells and in the upper respiratory tracts of hamsters. N501Y does not influence the binding and neutralization of most antibodies [30, 31].

### 2.6 E484K

The E484K mutation refers to a specific change in the spike protein of the SARS‐CoV‐2 virus, which is the virus that causes COVID‐19. This mutation involves a substitution of the amino acid at position 484 of the spike protein [43].

The E484K mutation is of interest to researchers and public health officials because it has been associated with potential immune escape. This means that it may affect the ability of the immune system, particularly antibodies, to recognize and neutralize the virus. This mutation has been found in several VOC and VOI, including some of the Beta (B.1.351), Gamma (P.1), and Theta (P.3) variants (Table 3) [44].

It is important to note that the situation with SARS‐CoV‐2 variants is dynamic, and ongoing research is conducted to understand the impact of specific mutations on the virus's transmissibility, severity of illness, and vaccine effectiveness. Public health agencies closely monitor the emergence and spread of variants to adapt strategies and recommendations accordingly [30, 31].

### 2.7 N‐Terminal Domain (NTD) Mutations

The NTD mutations refer to changes in the N‐terminal region of the spike protein of the SARS‐CoV‐2 virus. The spike protein is a key target for vaccines and antibodies because it plays a crucial role in the virus's ability to enter human cells. The NTD is one of the regions within the spike protein. Mutations in the NTD have been identified in various SARS‐CoV‐2 variants. These mutations can impact the virus's transmissibility, immune response, and possibly vaccine effectiveness. The NTD mutations have been a focus of attention as they may contribute to the virus's ability to evade neutralization by antibodies [44].

NTD deletions are present in several VOCs and VOIs and have also been reported commonly in persons with prolonged SARS‐CoV‐2 infections. Deletions at positions 69–70 appear to be associated primarily with increased virus replication, whereas those between positions 141–146 and 242–244 interfere with the NAc of NTD‐binding antibodies [32].

### 2.8 Mutations Close to the S1/S2 Furin Cleavage Site

Numerous SARS‐CoV‐2 variants have independently developed mutations just upstream of the polybasic S1/S2 furin cleavage, such as Q675H/R, Q677H/P, N679K, and P681H/R. P681H can be found in multiple additional SARS‐CoV‐2 lineages as well as the Alpha VOC and Theta VOI [45]. The Delta VOC and the Kappa VOI contain P681R. Both P681H and P681R have higher positive charges, which may affect virus tropism by causing S1/S2 cleavage in human airway epithelial cells (Table 2) [46].

**Table 2:** Summary of mMutations in emerging SARS‐Cov‐2 variants.

Region	Protein	Mutation	Remark	Reference
Spike	Spike glycoprotein	D614G	Increase the infectivity and viral load	[48]
Spike (Furin cleavage site)	Spike glycoprotein	P681R	Augments the viral infectivity	[49]
Spike (RBD region)	Spike glycoprotein	N501Y	Increase viral infectivity and the binding affinity to human ACE2 receptor	[50]
	Spike glycoprotein	E484K	Bind with monoclonal antibodies for reduction in antibody neutralization	
ORF1ab	NSP1	Deletion	Excess mutation and immune evasion	[51]
ORF8	ORF8	Deletion	Causing for milder viral infection	[52]

### 2.9 Nonspike Mutations

While much attention is often focused on the spike protein of the SARS‐CoV‐2 virus due to its significance in viral entry into host cells and its role in immunity and vaccines, the virus has other regions with genetic mutations as well. Non‐spike mutations occur throughout the SARS‐CoV‐2 genome, and some of these mutations may impact the virus's characteristics, such as transmissibility and severity of illness. It has been observed that mutations outside of the spike protein increase the transmissibility of SARS‐CoV‐2 by impeding the host's reaction to type I interferon. An enhanced transcription regulatory sequence was discovered to be introduced upstream of Orf9b, an interferon antagonist gene expressed as an alternative reading frame within the nucleocapsid coding region, due to a D3L mutation in the Alpha variant nucleocapsid gene [47].

## 3 Epidemiology for Variant of SARS‐CoV‐2

### 3.1 Epidemiology of Alpha (B.1.1.7 Lineage) Variant

The WHO has designated the SARS‐CoV‐2 variant of the B.1.1.7 lineage, also known as 20I/501Y.V1 or VOC 202012/01, as an Alpha variant. It was initially found in Kent, UK, and over time it spread throughout Europe and became the dominant strain in the UK. Three nations—Denmark, Switzerland, and the United States—saw an increase in transmission, which ranged from 59% to 74%. In the UK, patients infected with the Alpha variant were responsible for two‐thirds of higher death cases when compared to the original strains [53]. Nearly 95% of SARS‐CoV‐2 infections in the UK were caused by the Alpha variant as of March 29, 2021, and it had spread to 114 other countries [53]. As collected data of 3700 COVID‐19 patients from April 2020 to March 2021 in Tokat, Turkey, indicated; 30% were infected with the Alpha variants (Table 1) [54, 55].

Increased transmission over earlier virus lineages is thought to have contributed to the emergence of the B.1.1.7 (alpha variant), according to epidemiological studies. We compared the airborne transmission kinetics of B.1.1.7 with those of the prototype lineage alphavirus [56], to examine whether changes in transmission potential at the individual level account for the increased transmission potential of B.1.1.7 at the population level. The increased transmission may be explained by the B.1.1.7 spike's higher affinity for human ACE2 [57]. Tyrosine replaces the asparagine residue at position 501 of the B.1.1.7 spike RBD, enabling greater interactions with ACE2 residues through hydrogen bonding and the stacking of aromatic side chains, leading to a higher affinity binding to human ACE2 [58].

Comparing the Alpha lineage to earlier SARS‐CoV‐2 strains, it was reported that 23 genetic mutations were present. The Alpha variant carried mutations in the S gene, including N501Y, P681H, and the D614G mutation [59]. Particularly, the N501Y mutations gave the SARS‐CoV‐2 variant high infectivity and rapid transmissibility [60].

### 3.2 Epidemiology of Beta (B.1.351 Lineage) Variant

The second wave of SARS‐CoV‐2 infection was started by the discovery of the emergent SARS‐CoV‐2 variant of the B.1.351 lineage (also known as 501Y.V2) in early October 2020 in South Africa. The WHO dubbed this variant the Beta variant. As a result, the beta variant spread quickly throughout numerous nations and was quickly identified. Patients with the COVID‐19 infection experienced reinfection due to the beta variant [61, 62].

By the end of 2020, B.1.351 had spread to South Korea, Botswana, France, Scotland, Sweden, Switzerland, and the United Kingdom. Furthermore, by January 2021, the variant was identified in eight countries outside of the European Union (EU) (Australia, Brazil, Canada, China, Japan, Taiwan, United States [US], and Zambia) as well as eight countries inside the EU (Austria, Belgium, Denmark, Finland, Germany, Ireland, the Netherlands, and Norway). The World Health Organization reported B.1.351 cases in 41 countries [63].

About 570 B.1.351 cases had been confirmed globally as of January 19, 2021, with 447 of those cases occurring in South Africa. Affected nations outside of South Africa have between one and six confirmed cases; the UK stands out as an anomaly with 54 cases. The majority of cases in the EU have to do with travel, though not all of them involve previous trips to South Africa. Since each jurisdiction uses a different method for testing from its pool of all SARS‐CoV‐2‐positive specimens, the number of B.1.351 cases identified globally is probably underestimated [64].

As of February 2, 2021, two states in the US—South Carolina (*n* = 2) and Maryland (*n* = 1)—had reported three cases of B.1.351.8 As of February 1, 2021, seven B.1.351 cases had been reported in Alberta, four in British Columbia, and one in Ontario. As of January 23, 2021, there had been one case reported in Ontario [65].

The primary K417N, E484K, N501Y, and D614G mutations are covered by the beta variant's mutations; of these, the E484K mutation was able to lessen the variants' susceptibility to the potency of antibodies triggering immune escape [66]. It is noteworthy that the beta variant's nucleotide substitution of G23012A contributed to the E484K mutation, which is thought to have an impact on the antigenicity of the virus (Tables 1 and 3) [67].

**Table 3:** Summary of Variants of Interest (VOIs) of SARS‐CoV‐2 based recommended nomenclature from the World Health Organization (WHO) May 31, 2021 [33].

WHO name	Geographic region of first detection	Date first detected	Date of designation	Scientific name (pango lineage)
Epsilon variant	USA	March, 2020	March 5, 2021	B.1.427/B.1.429
Zeta variant	Brazil	April, 2020	March 17, 2021	P.2
Eta variant	Multiple countries	December, 2020	March 17, 2021	B.1.525
Theta variant	Philippines	January, 2021	March 24, 2021	P.3
Iota variant	USA	November, 2020	March 24, 2021	B.1.526
Kappa variant	India	October,2020	April 4, 2021	B.1.617.1

### 3.3 Epidemiology of Gamma (P.1 Lineage) Variant

During a routine screening at Tokyo's airport in January 2021, four travelers from Brazil were found to have the B.1.1.28.1 linage, also referred to as P.1, 20 J/501Y.V3 or the Gamma variant. Patients infected with the Gamma variant are more contagious than those with non‐P.1 strains because viral loads were almost ten times higher in the former. More than 51.1% of COVID‐19 patients identified in Umbria, Italy up until February 2021 had the SARS‐CoV‐2 P.1 variant. Furthermore, the WHO released epidemiological data that showed the emergence of the Gamma variant in over 45 countries up until March 30, 2021, including the US, Spain, Bangladesh, Uruguay, and Italy [68].

The Gamma variant was detected more frequently in São Paulo State in March 2021, with detection rates of 91.7% in the second week and 78.6% in the first. Other variations, such as P.2, B.1.1.7, and B.1.1.28, displayed a low frequency at the same time. This occurred here in March and April, with the Gamma lineage accounting for 75% and 89% of all sequences, respectively [69].

The study's findings, which were obtained in southern Brazil between the end of January and the end of February 2021, indicated the approximate proportion rate of Gamma and P.2 lineages. But only 33% of sequences in March belonged to the P.2 lineage, and in April, even more sequences belonged to the Gamma lineage. It has previously been reported that the Gamma lineage is capable of evading Nabs obtained through immunization or infection with viral variants that were previously in circulation. Furthermore, it was proposed that the Gamma is more transmissible than lineages that were previously in circulation [70]

The S protein of the gamma variant contained five mutations: K417T, E484K, N501Y, D614A, D614G, and H655Y substitutions. One noteworthy spike mutation that was discovered in early March 2020 [71] is D614G. The high viral viability of this mutation is most likely caused by minute changes in partitioning energy. Before March, 10% of all sequences worldwide had D614G. From April 1 to March 31, 2020, 67% of sequences had D614G mutations detected. From April 1 to May 18, 2020, 78% of sequences had D614G mutations detected. The advantage in replication efficiency observed over D614A, which can raise the likelihood of human‐to‐human transmission, is likely connected to the fast global spread and dominance of sequences carrying the D614G mutation. Three people who were returning to Korea from Uzbekistan in July 2020 had been infected by the D614G to D614A changed strains [40].

The Gamma variant's increasing affinity for binding to ACE2 is stronger than that of the Alpha variant, making it stronger than the Beta. This increased transmissibility of the virus allowed it to establish itself as the predominant strain in the areas where it arrived. The Gamma variant's increasing affinity for binding to ACE2 is stronger than that of the Alpha but still somewhat equal to the Beta, allowing for the augmentation of viral transmissibility and the potential for them to become primary strain in the areas where it arrived (Table 1 & 3) [72].

### 3.4 Epidemiology of Delta (B.1.617.2 Lineage) Variant

The variant known as SARS‐CoV‐2 Delta (B.1.617.2) was initially identified in India in December 2020. Then, in some areas, the delta variant has quickly emerged as the prevalent variant. By February 10, 2022, the United States had reported almost 1.4 million cases of the SARS‐CoV‐2 Delta variant. In late 2020, the SARS‐CoV‐2 Delta variant (B.1.617.2) was initially identified in India. The number of cases of the SARS‐CoV‐2 Delta variant worldwide as of February 10, 2022, broken down by nation or territory, is shown in this statistic [73].

WHO recently declared that viruses belonging to lineage B.1.617 have been classified as VOI or VOC. Three sub‐lineages make up B.1.617: B.1.617.1 (also called Kappa), B.1.617.2, and B.1.617.3. B.1.617. Two lineages have been separated into VOCs, which could have an impact on the virus's pathogenicity to humans, sensitivity to vaccination, and capacity to spread. The VOIs belonging to the B.1.617.1 and B.1.617.3 lineage are noteworthy variations that necessitate additional scrutiny [74].

An additional risk of infection arises from indirect contact with items used on the infected person or surfaces in the immediate surroundings. Pregnancy, ageing (particularly advanced age), various illnesses (such as asthma, COPD, hypertension, immune‐compromised states from blood or bone marrow transplants, HIV), smoking, and diabetes are listed as the main risk factors for this newly discovered viral disease [75].

The delta variant is more transmissible than the alpha variant, according to available data. The ratio, which ranges between 40% and 60%, might indicate a higher risk of hospitalization. The existence of D614G in the Delta genome, along with more mutations and closer synergy, may be the causes of the recent, sharp increase in cases linked to the Delta. Delta, the predominant virus strain in 2020, is well recognized for its potent capacity for replication and transmission [76].

Furthermore, Guangzhou epidemiological studies revealed that patients with the Delta variant could spread quickly even if people avoided talking to each other when using the loo or eating in the same area (Tables 1 and 3) [77].

### 3.5 Epidemiology of Omicron (B.1.1.529 Lineage) Variant

The B.1.1.529 variant was initially discovered in South Africa and Botswana in November 2021. At least 35 mutations in the spike protein were discovered to be present in this variant, and rising cases in South Africa were in line with the identification of the B.1.1.529 variant [78]. The B.1.1.529 variant was named the Omicron variant by the WHO, who classified it as a new VOC. Over 50% of the world's population is expected to contract the Omicron variant. In the 23 months since the first case of COVID‐19 was reported, at least 260 million SARS‐CoV‐2 infections and 5.2 million deaths globally have been recorded [79].

By March 31, 2022, 99.7% of sequences submitted between February 23 and March 24, 2022, were submitted with the Omicron variant, which had been found in 188 countries and had already established itself as the predominant strain worldwide. Four sublineages have split off from the Omicron variant: BA.1, BA.1.1, BA.2, and BA.3. The majority of Omicron variants in circulation are BA.1, BA.1.1, and BA.2. The original form, or Omicron BA.1 variant, can be distinguished by S‐gene target failure (SGTF). The R346K mutation in the Omicron BA.1.1 variant makes it a subvariant of BA.1. The Omicron BA.2 variant has become dominant in several countries, including Denmark, India, Norway, and Singapore, suggesting it may have a selective advantage over the Omicron BA.1 variant. Notably, the proportion of BA.2, which cannot cause SGTF, has been rising. According to a Danish epidemiological study, BA.2's effective reproduction number was 1.26 times higher than BA.1's [80, 81].

Omicron and Delta case epidemiology in Wales, November 29, 2021–December 14, 2021 The first 1000 Omicron cases were discovered during the same time period as 8168 Delta cases.

The latest omicron variant is XBB.15. The prevalence of XBB.1.5 started to increase in late 2022, reaching a peak of 55% in week 12 of 2023 after reaching a prevalence of 3% in week 51 of 2022. The US Centers for Disease Control and Prevention report that XBB.1.5 is responsible for 40.5% of COVID‐19 infections in the US as of latest report. The virus has spread quickly; when it was initially discovered in the US in late September of 2022, it accounted for 0.1% of all cases [82, 83].

According to CDC latest report, the status for most variants is under the variants being monitor except, variants containing the F456L spike mutations and B.1.1.529 and descendant lineages or Omicron (parent lineages) [81].

Cardiff's local authority was the site of the first confirmed cases of Omicron, and by December 14, the city had more than 60 cases of Omicron for every 100,000 people. On the other hand, at this time, Wrexham, North Wales, had the highest rates of the Delta variant (including VUI‐21OCT‐01, AY.4.2) per 100,000 people, with over 400 cases per 100,000 people [84].

48 h after infection, the variation also reaches higher levels in the tissue than delta, according to researchers at the University of Hong Kong. Additionally, Omicron may be more contagious than delta, according to Garcia‐Beltran and associates [85]. It seemed that the symptoms were comparable to those of other coronavirus variations. The Omicron coronavirus variant is still spreading globally, causing symptoms in infected individuals such as runny nose, headache, fatigue (either moderate or severe), sneezing, and sore throat. To ascertain how the variant will impact the population, it is helpful to consider factors such as immune evasion, the rate of transmission, and the percentages of individuals who develop severe illnesses and pass away [86].

Moreover, the Omicron variant of SARS‐CoV‐2 was predominant in South Africa. Additionally, about 37 major mutations in the S protein were found to be present in the Omicron variant. These mutations, which help viruses evade antibody neutralization, include K417N, S477N, Q498R, E484A, and N501Y. As a result, the Omicron variant may be more visible in terms of immune escape. It is noteworthy that the insertion mutation (ins214EPE), which was expressed in seasonal coronaviruses (e.g., HCoV‐229E), was initially discovered in the Omicron variant and had not previously been detected in any SARS‐CoV‐2 strains [87, 88, 89].

### 3.6 Implications of New Variants of SARS‐CoV‐2 on the Pathogenesis of COVID‐19

Variations in the genetic makeup of the SARS‐CoV‐2 virus may have an impact on the virus's pathogenesis, particularly if they affect the RBD, a crucial target for monoclonal antibodies derived from vaccine sera and a mechanism through which the virus enters host cells [8]. All five of the known VOCs—Alpha (B.1.1.7), Beta (B.1.351), Gamma (P.1), Delta (B.1.617.2), and Omicron (B.1.1.529)—have mutations in the RBD and NTD. Of these, the RBD variants all share the N501Y mutation, which increases the spike protein's affinity for ACE 2 receptors, thereby facilitating the viral attachment and subsequent host cell entry. In addition to NBD, RBD functions as the primary target for neutralization and promotes the production of antibodies in response to antisera or vaccinations [90, 91]. It's interesting to note that compared to N501Y‐RBD and ACE2, the binding affinities of the B.1.351 variant and P.1 variant with mutations N417/K848/Y501‐RBD and ACE2 were substantially lower [92, 93, 94].

## 4 Vaccines for SARS‐CoV‐2

There are two varieties of COVID‐19 vaccinations that are approved or licensed. Multiple licensed, approved, recommended, and age‐appropriate COVID‐19 vaccinations are available. None is recommended over the others.

### 4.1 mRNA SARS‐CoV‐2 Vaccines

mRNA vaccines instruct our cells on how to produce a protein, or even just a portion of a protein, that elicits an immune response within our bodies using mRNA that has been synthesized in a lab. Within a few days of immunization, the vaccine's mRNA is broken down and excreted from the body. Pfizer‐BioNTech and Moderna COVID‐19 vaccines are mRNA vaccines [95].

### 4.2 Protein Subunit SARS‐CoV‐2 Vaccines

There are also COVID‐19 vaccines made from protein subunits. The COVID‐19 vaccine made of protein subunits is called Novavax. Vaccines using protein subunits comprise fragments of the COVID‐19 virus. The viral fragments are from spike protein of COVID‐19. Another component known as an adjuvant is included in the COVID‐19 vaccination Novavax. It facilitates the immune system's reaction to that protein surge. The immune system may swiftly react to the virus's true spike protein once it has learned how to react to the spike protein, shielding you against COVID‐19 [95].

The COVID‐19 pandemic has necessitated quick response times and historically short vaccine development timelines. WHO reports that 195 vaccine candidates are being studied in preclinical development, and 147 vaccine candidates based on various platforms are presently being assessed in human clinical trials [16, 95, 96, 97].

The COVID‐19 four vaccines have been approved and are being used globally, despite the pandemic having only been going for slightly over 18 months: Pfizer/BioNtech's BNT162b2, Moderna's mRNA‐1273, AstraZeneca's ChAdOx1, and Janssen's Ad26.COV2S. The FDA has authorized the use of the Pfizer/BioNtech, Moderna, and Janssen vaccines for emergency situations, while the AstraZeneca vaccine is widely accepted outside of the United States [98, 99, 100]. Thus far, 2,794,490 doses of the COVID‐19 vaccine (produced by Johnson & Johnson, Sinopharm, or AstraZeneca have been given in Ethiopia. 752,888 (27%) of the vaccinated individuals have received all recommended vaccinations has necessitated quick response times and historically short vaccine development timelines WHO reports that 195 vaccine candidates are being studied in preclinical development, and 147 vaccine candidates based on various platforms are presently being assessed in human clinical trials (Table 4) [14, 91, 93, 101, 102].

**Table 4:** Characteristics of the principal SARS CoV‐2 vaccines [20, 102, 103, 104].

Vaccine	Target variants	Antigen	Efficacy	Mechanism of action	Phase
Pfizer/BioNTtech	Alpha, Alpha with E484K, Beta, B.1.526, B.1.617, Gamma, Delta, Delta plus, Lambda and B.1.1.519 lineages	Full‐length spike protein	89.5%–93.7%(Alpha);75%–100% (Beta); 52.4%–88% (Delta); 22.5% (Omicron);89%–96% (for pregnant women)	mRNA	Phase 3 trial (adults); Phase 2/3 trial (children)
Moderna	SARS‐CoV‐2 or variants	Segments of SARS‐CoV‐2 hereditary material	94.1%; 96.4% (Beta); 89% (Alpha); 85% (Beta, Gamma, B.1.617); 50.6% (Delta)	mRNA	Phase 3 trial
Astra‐zeneca	SARS‐CoV‐2 or variants	Whole‐length S protein	62.1–79%; 61.1% (Beta); 10.4% (Beta; HIV‐negative); 74.5% (Alpha); 67% (Delta)	Adenovirus viral vector (replication deficient chimpanzee adenovirus)	Phase 3 trial
Janssen Johnson & Johnson/Ad26.COV2.S vaccine	D614G, Beta, P.2 lineage	Whole S protein of SARS‐CoV‐2	66.9–76.7% (14 days); 66.1–85.4% (28 days); 72% (Alpha); 57% (Beta, Gamma, B.1.617)	Adenovirus viral vector (replication deficient chimpanzee adenovirus)	Phase 3 trial

## 5 Conclusion and Recommendation

Numerous VOCs, including Alpha (B.1.1.7), Beta (B.1.351), Gamma (P.1), Delta (B.1.617.2), and Omicron (B.1.1.529), have been found and reported globally since the start of the COVID‐19 pandemic. CoV‐2 can spread roughly twice as quickly as the original strain thanks to the delta variant and its offspring, and the disease's severity may also increase. However, since the Omicron variant first surfaced on November 26, 2021, concerns regarding its transmissibility, virulence, infectivity, and immune responses have grown. As of right now, the frequency of B.1.1.529 (Omicron) is increasing; on December 13, 2021, it accounted for 5% of all SARS‐CoV‐2 cases. This variant is noteworthy due to its potential for increased transmissibility.

Because the current vaccines have different variants, they are effective against hospitalization and severe disease. Effective ways to stop the spread of the various variants include accelerating vaccination awareness, raising the coverage rate, and putting intervention strategies like mask wear into practice. On the other hand, vaccination against SARS‐CoV‐2 on its own, in the absence of intervention measures, may result in ongoing transmission and the emergence of new variants.

## Author Contributions

**Mulat Erkihun:** conceptualization, investigation, writing–original draft, writing–review and editing, methodology, data curation. **Bayu Ayele:** conceptualization, writing–review and editing, visualization, validation. **Zelalem Asmare:** conceptualization, writing–review and editing, methodology, validation, visualization. **Kirubel Endalamaw:** writing–original draft, investigation, methodology, data curation.

## Ethics Statement

The authors have nothing to report.

## Conflicts of Interest

The authors declare no conflicts of interest.

## Data Availability

Data sharing is not applicable to this article as no new data were created or analyzed in this study. All authors have read and approved the final version of the manuscript [corresponding author or manuscript guarantor] had full access to all of the data in this study and takes complete responsibility for the integrity of the data and the accuracy of the data analysis.

## References

[1] C. Wang , P. W. Horby , F. G. Hayden , and G. F. Gao , “A Novel Coronavirus Outbreak of Global Health Concern,” Lancet 395, no. 10223 (2020): 470–473. 31986257 10.1016/S0140-6736(20)30185-9 PMC7135038

[2] N. Zhu , D. Zhang , W. Wang , et al., “A Novel Coronavirus From Patients With Pneumonia in China, 2019,” New England Journal of Medicine 382 (2020): 727–733. 31978945 10.1056/NEJMoa2001017 PMC7092803

[3] Worldometer . COVID‐19 Coronavirus Pandemic 2022 (2022), https://www.worldometers.info/coronavirus/.

[4] T. Tolossa , E. Merdassa Atomssa , G. Fetensa , et al., “Acute Respiratory Distress Syndrome Among Patients With Severe COVID‐19 Admitted to Treatment Center of Wollega University Referral Hospital, Western Ethiopia,” Plos One 17, no. 6 (2022): e0267835. 35709142 10.1371/journal.pone.0267835 PMC9202843

[5] T. Kaizek , D. Erdman , C. Goldsmith , S. Zaki , and T. Peret , “A Novel Coronavirus Associated With Sever Acute Respiratory Syndrome,” New England Journal of Medicine 348 (2003): 1953–1966. 12690092 10.1056/NEJMoa030781

[6] J. F. Chan , K.‐H. Kok , Z. Zhu , et al., “Genomic Characterization of the 2019 Novel Human‐Pathogenic Coronavirus Isolated From a Patient With Atypical Pneumonia After Visiting Wuhan,” Emerging Microbes & Infections 9, no. 1 (2020): 221–236. 31987001 10.1080/22221751.2020.1719902 PMC7067204

[7] S. Chakraborty , “Evolutionary and Structural Analysis Elucidates Mutations on SARS‐CoV2 Spike Protein With Altered Human ACE2 Binding Affinity,” Biochemical and Biophysical Research Communications 538 (2021): 97–103. 33602511 10.1016/j.bbrc.2021.01.035 PMC7883683

[8] R. Varghese , G. Digholkar , J. Karsiya , et al., “PDE5 Inhibitors: Breaking New Grounds in the Treatment of COVID‐19,” Drug Metabolism and Personalized Therapy (2023). 10.1515/dmdi-2023-0011 37608528

[9] P. J. M. Brouwer , T. G. Caniels , K. van der Straten , et al., “Potent Neutralizing Antibodies From COVID‐19 Patients Define Multiple Targets of Vulnerability,” Science 369, no. 6504 (2020): 643–650. 32540902 10.1126/science.abc5902 PMC7299281

[10] C. E. Gómez , B. Perdiguero , and M. Esteban , “Emerging SARS‐CoV‐2 Variants and Impact in Global Vaccination Programs Against SARS‐CoV‐2/COVID‐19,” Vaccines 9, no. 3 (2021): 243. 33799505 10.3390/vaccines9030243 PMC7999234

[11] R. K. Mohapatra , R. Tiwari , A. K. Sarangi , et al., “Twin Combination of Omicron and Delta Variant Triggering a Tsunami Wave of Ever High Surges in COVID‐19 Cases: A Challenging Global Threat With a Special Focus on Indian Sub‐Continent,” Journal of Medical Virology 94 (2022): 1761. 35014038 10.1002/jmv.27585 PMC9015634

[12] WHO , *Tracking SARS‐CoV‐2 Variants* (WHO, 2022), https://www.who.int/.

[13] CDC , *SARS‐CoV‐2 Variant Classifications and Definitions [Internet]* (CDC, 2021), https://www.cdc.gov/coronavirus/.

[14] Ephi , *COVID‐19 PANDEMIC PREPAREDNESS AND RESPONSE IN ETHIOPIA* (Ephi, 2021), https://ephi.gov.et/.

[15] M. Alkhatib , R. Salpini , L. Carioti , et al., “Update on SARS‐CoV‐2 Omicron Variant of Concern and its Peculiar Mutational Profile,” Microbiology Spectrum 10, no. 2 (2022): e0273221. 35352942 10.1128/spectrum.02732-21 PMC9045195

[16] R. Rana , R. Kant , T. Kumra , S. Gupta , D. S. Rana , and N. K. Ganguly , “An Update on SARS‐CoV‐2 Immunization and Future Directions,” Frontiers in Pharmacology 14 (2023): 1125305. 36969857 10.3389/fphar.2023.1125305 PMC10033701

[17] P. A. Ortiz‐Pineda and C. H. Sierra‐Torres , “Evolutionary Traits and Genomic Surveillance of SARS‐CoV‐2 in South America,” Global Health 2022 (2022): 1–9. 10.1155/2022/8551576 PMC9132712 35655960

[18] T. G. Ksiazek , D. Erdman , C. S. Goldsmith , et al., “A Novel Coronavirus Associated With Severe Acute Respiratory Syndrome,” New England Journal of Medicine 348, no. 20 (2003): 1953–1966. 12690092 10.1056/NEJMoa030781

[19] Q. Fernandes , V. P. Inchakalody , M. Merhi , et al., “Emerging COVID‐19 Variants and Their Impact on SARS‐CoV‐2 Diagnosis, Therapeutics and Vaccines,” Annals of medicine 54, no. 1 (2022): 524–540. 35132910 10.1080/07853890.2022.2031274 PMC8843115

[20] I. A. Charitos , A. Ballini , R. Lovero , et al., “Update on COVID‐19 and Effectiveness of a Vaccination Campaign in a Global Context,” International Journal of Environmental Research and Public Health 19, no. 17 (2022): 10712. 36078427 10.3390/ijerph191710712 PMC9518080

[21] S. Cherian , V. Potdar , S. Jadhav , et al., “SARS‐CoV‐2 Spike Mutations, L452R, T478K, E484Q and P681R, in the Second Wave of COVID‐19 in Maharashtra, India,” Microorganisms 9, no. 7 (2021): 1542. 34361977 10.3390/microorganisms9071542 PMC8307577

[22] J. R. Fauver , M. E. Petrone , E. B. Hodcroft , et al., “Coast‐to‐Coast Spread of SARS‐CoV‐2 During the Early Epidemic in the United States,” Cell 181, no. 5 (2020): 990–996.e5. 32386545 10.1016/j.cell.2020.04.021 PMC7204677

[23] B. Korber , W. M. Fischer , S. Gnanakaran , et al., “Tracking Changes in SARS‐CoV‐2 Spike: Evidence That D614G Increases Infectivity of the COVID‐19 Virus,” Cell 182 (2020): 812–827.e19. 32697968 10.1016/j.cell.2020.06.043 PMC7332439

[24] N. Oreshkova , R. J. Molenaar , S. Vreman , et al., “SARS‐CoV‐2 Infection in Farmed Minks, the Netherlands, April and May 2020,” Eurosurveillance 25, no. 23 (2020): 2001005. 32553059 10.2807/1560-7917.ES.2020.25.23.2001005 PMC7403642

[25] K. Alves , J. S. Plested , S. Galbiati , et al., “Immunogenicity and Safety of a Fourth Homologous Dose of NVX‐CoV2373,” Vaccine 41, no. 29 (2023): 4280–4286. 37271706 10.1016/j.vaccine.2023.05.051 PMC10237325

[26] World Health Organization , *Weekly Epidemiological Update on COVID‐19* (World Health Organization, 2021), 44:1–26, https://www.who.int/.

[27] D. V. Parums , “Revised World Health Organization (WHO) Terminology for Variants of Concern and Variants of Interest of SARS‐CoV‐2,” Medical Science Monitor: International Medical Journal of Experimental and Clinical Research 27 (2021): e933622‐1. 34149046 10.12659/MSM.933622 PMC8230247

[28] D. A. Collier , A. De Marco , I. A. T. M. Ferreira , et al., “Sensitivity of SARS‐CoV‐2 B. 1.1. 7 to mRNA Vaccine‐Elicited Antibodies,” Nature 593, no. 7857 (2021): 136–141. 33706364 10.1038/s41586-021-03412-7 PMC7616976

[29] D. Ho , P. Wang , L. Liu , et al., Increased Resistance of SARS‐CoV‐2 Variants B. 1.351 and B. 1.1. 7 to Antibody Neutralization *Research Square [Preprint]* (2021): rs.3.rs‐155394, 10.21203/rs.3.rs-155394/v1.

[30] P. Wang , R. G. Casner , M. S. Nair , et al., “Increased Resistance of SARS‐CoV‐2 Variant P. 1 to Antibody Neutralization,” Cell Host & Microbe 29, no. 5 (2021): 747–751. 33887205 10.1016/j.chom.2021.04.007 PMC8053237

[31] R. Challen , L. Dyson , C. E. Overton , et al., Early Epidemiological Signatures of Novel SARS‐CoV‐2 Variants: Establishment of B. 1.617. 2 in England (MedRxiv, 2021).

[32] K. Kanatzhan , Y. Zhanar , N. Gulnaz , et al., “The Omicron Strain of Coronavirus May Be More Transmissible Than Other Variants and Is Partially Resistant to Existing Vaccines,” NVEO‐NATURAL VOLATILES & ESSENTIAL OILS Journal| NVEO 8 (2021): 13696–13706.

[33] World Health Organization , *Tracking SARS‐CoV‐2 Variants* (World Health Organization, 2021), https://www.who.int/.

[34] K. Sobhani , S. Cheng , R. A. Binder , et al., “Clinical Utility of SARS‐CoV‐2 Serological Testing and Defining a Correlate of Protection,” Vaccines 11, no. 11 (2023): 1644. 38005976 10.3390/vaccines11111644 PMC10674881

[35] G. Alhamid , H. Tombuloglu , A. A. Rabaan , and E. Al‐Suhaimi , “SARS‐CoV‐2 Detection Methods: A Comprehensive Review,” Saudi Journal of Biological Sciences 29, no. 11 (2022): 103465. 36186678 10.1016/j.sjbs.2022.103465 PMC9512523

[36] W. Jiang , W. Ji , Y. Zhang , et al., “An Update on Detection Technologies for SARS‐CoV‐2 Variants of Concern,” Viruses 14, no. 11 (2022): 2324. 36366421 10.3390/v14112324 PMC9693800

[37] Q. Huang , H. Qiu , P. W. Bible , et al., “Early Detection of SARS‐CoV‐2 Variants Through Dynamic Co‐Mutation Network Surveillance,” Frontiers in public health 11 (2023): 1015969. 36755900 10.3389/fpubh.2023.1015969 PMC9901361

[38] P. de Sequera , B. Quiroga , and M. Goicoechea , “Actualización de las recomendaciones de medidas de prevención y aislamiento frente al SARS‐CoV‐2 en las unidades de diálisis: un posicionamiento de la Sociedad Española de Nefrología,” Nefrología 42, no. 6 (2022): 714–721. 10.1016/j.nefro.2022.10.001 PMC9554317 36247494

[39] K. Tao , P. L. Tzou , J. Nouhin , et al., “The Biological and Clinical Significance of Emerging SARS‐CoV‐2 Variants,” Nature Reviews Genetics 22, no. 12 (2021): 757–773. 10.1038/s41576-021-00408-x PMC8447121 34535792

[40] J. A. Plante , Y. Liu , J. Liu , et al., “Spike Mutation D614G Alters SARS‐CoV‐2 Fitness,” Nature 592, no. 7852 (2021): 116–121. 33106671 10.1038/s41586-020-2895-3 PMC8158177

[41] Y. J. Hou , S. Chiba , P. Halfmann , et al., “SARS‐CoV‐2 D614G Variant Exhibits Efficient Replication Ex Vivo and Transmission In Vivo,” Science 370, no. 6523 (2020): 1464–1468. 33184236 10.1126/science.abe8499 PMC7775736

[42] X. Shen , H. Tang , C. McDanal , et al., “SARS‐CoV‐2 Variant B. 1.1. 7 Is Susceptible to Neutralizing Antibodies Elicited by Ancestral Spike Vaccines,” Cell Host & Microbe 29, no. 4 (2021): 529–539. 33705729 10.1016/j.chom.2021.03.002 PMC7934674

[43] T. Tada , B. M. Dcosta , M. I. Samanovic , et al., “Convalescent‐Phase Sera and Vaccine‐Elicited Antibodies Largely Maintain Neutralizing Titer Against Global SARS‐CoV‐2 Variant Spikes,” MBio 12, no. 3 (2021): e00696‐21. 34060334 10.1128/mBio.00696-21 PMC8262901

[44] K. R. McCarthy , L. J. Rennick , S. Nambulli , et al., “Recurrent Deletions in the SARS‐CoV‐2 Spike Glycoprotein Drive Antibody Escape,” Science 371, no. 6534 (2021): 1139–1142. 33536258 10.1126/science.abf6950 PMC7971772

[45] E. B. Hodcroft , D. B. Domman , D. J. Snyder , et al., “Emergence in Late 2020 of Multiple Lineages of SARS‐CoV‐2 Spike Protein Variants Affecting Amino Acid Position 677,” MedRxiv (2021): [Preprint].

[46] B. A. Johnson , X. Xie , A. L. Bailey , et al., “Loss of Furin Cleavage Site Attenuates SARS‐CoV‐2 Pathogenesis,” Nature 591, no. 7849 (2021): 293–299. 33494095 10.1038/s41586-021-03237-4 PMC8175039

[47] M. D. Parker , B. B. Lindsey , D. R. Shah , et al., “Altered Subgenomic RNA Expression in SARS‐CoV‐2 B. 1.1. 7 Infections,” bioRxiv (2021): [preprint].

[48] W. Zhou , C. Xu , P. Wang , et al, “N439K Variant in Spike Protein Alter the Infection Efficiency and Antigenicity of SARS‐CoV‐2 Based on Molecular Dynamics Simulation,” Frontiers in Cell and Developmental Biology 9 (2021): 2071. 10.3389/fcell.2021.697035 PMC8369991 34414185

[49] S. Cherian , V. Potdar , S. Jadhav , et al., “SARS‐CoV‐2 Spike Mutations, L452R, T478K, E484Q and P681R, in the Second Wave of COVID‐19 in Maharashtra, India,” Microorganisms 9 (2021): 1542. 34361977 10.3390/microorganisms9071542 PMC8307577

[50] J. T. Ortega , B. Jastrzebska , and H. R. Rangel , “Omicron SARS‐CoV‐2 Variant Spike Protein Shows an Increased Affinity to the Human ACE2 Receptor: an in Silico Analysis,” Pathogens 11, no. 1 (2021): 45. 35055993 10.3390/pathogens11010045 PMC8779645

[51] S. Jangra , C. Ye , R. Rathnasinghe , et al., “SARS‐CoV‐2 Spike E484K Mutation Reduces Antibody Neutralisation,” Lancet Microbe 2, no. 7 (2021): e283–e284. 33846703 10.1016/S2666-5247(21)00068-9 PMC8026167

[52] R. Wang , J. Chen , Y. Hozumi , C. Yin , and G.‐W. Wei , “Decoding Asymptomatic COVID‐19 Infection and Transmission,” Journal of Physical Chemistry Letters 11, no. 23 (2020): 10007–10015. 33179934 10.1021/acs.jpclett.0c02765 PMC8150094

[53] D. J. Grint , K. Wing , E. Williamson , et al., “Case Fatality Risk of the SARS‐CoV‐2 Variant of Concern B. 1.1. 7 in England, 16 November to 5 February,” Eurosurveillance 26, no. 11 (2021): 2100256. 33739254 10.2807/1560-7917.ES.2021.26.11.2100256 PMC7976383

[54] M. Cetin , P. O. Balci , H. Sivgin , et al., “Alpha Variant (B. 1.1. 7) of SARS‐CoV‐2 Increases Fatality‐Rate for Patients Under Age of 70 Years and Hospitalization Risk Overall,” Acta Microbiologica et Immunologica Hungarica 68, no. 3 (2021): 153–161. 10.1556/030.2021.01524 34383706

[55] M. N. Zahan , H. Habibi , A. Pencil , et al., “Diagnosis of COVID‐19 in Symptomatic Patients: An Updated Review,” Vacunas 23, no. 1 (2022): 55–61. 34276268 10.1016/j.vacun.2021.06.002 PMC8275488

[56] N. G. Davies , S. Abbott , R. C. Barnard , et al. “Estimated Transmissibility and Severity of Novel SARS‐CoV‐2 Variant of Concern 12/01 in England,” Science 372, no. 6538 (2021): eabg3055. 33658326 10.1126/science.abg3055 PMC8128288

[57] D. Frampton , T. Rampling , A. Cross , et al., “Genomic Characteristics and Clinical Effect of the Emergent SARS‐CoV‐2 B. 1.1. 7 Lineage in London, UK: A Whole‐Genome Sequencing and Hospital‐Based Cohort Study,” Lancet Infectious Diseases 21, no. 9 (2021): 1246–1256. 33857406 10.1016/S1473-3099(21)00170-5 PMC8041359

[58] C. Laffeber , K. de Koning , R. Kanaar , and J. H. G. Lebbink , “Experimental Evidence for Enhanced Receptor Binding by Rapidly Spreading SARS‐CoV‐2 Variants,” Journal of Molecular Biology 433, no. 15 (2021): 167058. 34023401 10.1016/j.jmb.2021.167058 PMC8139174

[59] T.‐J. Yang , P.‐Y. Yu , Y.‐C. Chang , et al., “Effect of SARS‐CoV‐2 B. 1.1. 7 Mutations on Spike Protein Structure and Function,” Nature Structural & Molecular Biology 28, no. 9 (2021): 731–739. 10.1038/s41594-021-00652-z 34385690

[60] C. Graham , J. Seow , I. Huettner , et al., “Neutralization Potency of Monoclonal Antibodies Recognizing Dominant and Subdominant Epitopes on SARS‐CoV‐2 Spike Is Impacted by the B. 1.1. 7 Variant,” Immunity 54, no. 6 (2021): 1276–1289.e6. 33836142 10.1016/j.immuni.2021.03.023 PMC8015430

[61] S. Saha , A. M. Tanmoy , Y. Hooda , et al, “COVID‐19 Rise in Bangladesh Correlates With Increasing Detection of B. 1.351 Variant,” BMJ Specialist Journals 76 (2021): e006012. 10.1136/bmjgh-2021-006012 PMC8102859 33952579

[62] K. Xu , P. Gao , S. Liu , et al., “Protective Prototype‐Beta and Delta‐Omicron Chimeric RBD‐Dimer Vaccines Against SARS‐CoV‐2,” Cell 185, no. 13 (2022): 2265–2278.e14. 35568034 10.1016/j.cell.2022.04.029 PMC9042943

[63] World Health Organization , Weekly Epidemiological Update (World Health Organization, 2020).

[64] Assessment RR, Risk Related to the Spread of New SARS‐CoV‐2 Variants of Concern in the EU/EEA–First Update (European Centre for Disease Prevention and Control an Agency of the European Union, 2021), https://www.ecdc.eur/.

[65] D. Skowronski , COVID‐19 Situation Report Week 44 (British Columbia, Center for Disease Control, 2020).

[66] A. J. Greaney , A. N. Loes , K. Crawford , et al., “Comprehensive Mapping of Mutations in the SARS‐CoV‐2 Receptor‐Binding Domain That Affect Recognition by Polyclonal Human Plasma Antibodies,” Cell Host & Mmicrobe 29, no. 3 (2021): 463–476. 10.1016/j.chom.2021.02.003 PMC7869748 33592168

[67] X. Xie , Y. Liu , J. Liu , et al., “Neutralization of SARS‐CoV‐2 Spike 69/70 Deletion, E484K and N501Y Variants by BNT162b2 Vaccine‐Elicited Sera,” Nature Medicine 27, no. 4 (2021): 620–621. 10.1038/s41591-021-01270-4 33558724

[68] F. Di Giallonardo , I. Puglia , V. Curini , et al., “Emergence and Spread of SARS‐CoV‐2 Lineages B. 1.1. 7 and P. 1 in Italy,” Viruses 13, no. 5 (2021): 794. 33946747 10.3390/v13050794 PMC8146936

[69] G. R. Barbosa , L. V. L. Moreira , A. F. O. Justo , et al., “Rapid Spread and High Impact of the Variant of Concern P. 1 in the Largest City of Brazil,” Journal of Infection 83, no. 1 (2021): 119–145. 10.1016/j.jinf.2021.04.008 33865897

[70] F. G. Naveca , V. Nascimento , V. C. de Souza , et al., “COVID‐19 in Amazonas, Brazil, Was Driven by the Persistence of Endemic Lineages and P. 1 Emergence,” Nature Medicine 27, no. 7 (2021): 1230–1238. 10.1038/s41591-021-01378-7 34035535

[71] B. Korber , W. M. Fischer , S. Gnanakaran , et al., “Tracking Changes in SARS‐CoV‐2 Spike: Evidence That D614G Increases Infectivity of the COVID‐19 Virus,” Cell 182, no. 4 (2020): 812–827.e19. 32697968 10.1016/j.cell.2020.06.043 PMC7332439

[72] A. Gidari , S. Sabbatini , S. Bastianelli , et al., “Cross‐Neutralization of SARS‐CoV‐2 B. 1.1. 7 and P. 1 Variants in Vaccinated, Convalescent and P. 1 Infected,” Journal of Infection 83, no. 4 (2021): 467–472. 34320390 10.1016/j.jinf.2021.07.019 PMC8310664

[73] J. R. Lechien and S. Saussez , “Importance of Epidemiological Factors in the Evaluation of Transmissibility and Clinical Severity of SARS‐CoV‐2 Variants,” The Lancet Infectious Diseases 22, no. 1 (2022): 2–3. 10.1016/S1473-3099(21)00474-6 PMC8397299 34461055

[74] S. W. X. Ong , C. J. Chiew , L. W. Ang , et al., “Clinical and Virological Features of SARS‐CoV‐2 Variants of Concern: A Retrospective Cohort Study Comparing B. 1.1. 7 (Alpha), B. 1.315 (Beta), and B. 1.617. 2 (Delta),” Clinical Infectious Diseases 75, no. 1 (2022): e1128–e1136. 34423834 10.1093/cid/ciab721 PMC8522361

[75] A. Hafeez , S. Ahmad , S. A. Siddqui , M. Ahmad , and S. Mishra , “A Review of COVID‐19 (Coronavirus Disease‐2019) Diagnosis, Treatments and Prevention,” EJMO 4, no. 2 (2020): 116–125.

[76] E. Volz , V. Hill , J. T. McCrone , et al, “Evaluating the Effects of SARS‐CoV‐2 Spike Mutation D614G on Transmissibility and Pathogenicity,” cell 184, no. 1 (2021): 64–75. e11. 33275900 10.1016/j.cell.2020.11.020 PMC7674007

[77] M. Zhang , J. Xiao , A. Deng , et al., “Transmission Dynamics of an Outbreak of the COVID‐19 Delta Variant B. 1.617. 2—Guangdong Province, China, May–June 2021,” China CDC Weekly 3, no. 27 (2021): 584–586. 34594941 10.46234/ccdcw2021.148 PMC8392962

[78] S. K. Saxena , S. Kumar , S. Ansari , et al., “Characterization of the Novel SARS‐CoV‐2 Omicron (B. 1.1. 529) Variant of Concern and its Global Perspective,” Journal of Medical Virology 94, no. 4 (2022): 1738–1744. 34905235 10.1002/jmv.27524

[79] Y. Wang , Y. Long , F. Wang , C. Li , and W. Liu , “Characterization of SARS‐CoV‐2 Recombinants and Emerging Omicron Sublineages,” International Journal of Medical Sciences 20, no. 1 (2023): 151–162. 36619228 10.7150/ijms.79116 PMC9812801

[80] S. Chenchula , P. Karunakaran , S. Sharma , and M. Chavan , “Current Evidence on Efficacy of COVID‐19 Booster Dose Vaccination Against the Omicron Variant: A Systematic Review,” Journal of Medical Virology 94, no. 7 (2022): 2969–2976. 35246846 10.1002/jmv.27697 PMC9088621

[81] C. Yang , H. Zhao , C. P. Shannon , and S. J. Tebbutt , “Omicron Variants of SARS‐CoV‐2 and Long Covid,” Frontiers in Immunology 13 (2022): 1061686. 36569883 10.3389/fimmu.2022.1061686 PMC9780375

[82] K. Ito , C. Piantham , and H. Nishiura , Estimating Relative Generation Times and Relative Reproduction Numbers of Omicron BA. 1 and BA. 2 With Respect to Delta in Denmark (MedRxiv, 2022). [Preprint]. 10.3934/mbe.2022418 35942746

[83] S. Pather , A. Muik , R. Rizzi , and F. Mensa , “Clinical Development of Variant‐Adapted BNT162b2 COVID‐19 Vaccines: The Early Omicron Era,” Expert Review of Vaccines 22, no. 1 (2023): 650–661. 37417000 10.1080/14760584.2023.2232851

[84] N. Pacchiarini , C. Sawyer , C. Williams , et al., “Epidemiological Analysis of the First 1000 Cases of SARS‐CoV‐2 Lineage BA. 1 (B. 1.1. 529, Omicron) Compared With Co‐Circulating Delta in Wales, UK,” Influenza and Other Respiratory Viruses 16 (2022): 986–993. 35822273 10.1111/irv.13021 PMC9350272

[85] V. Sharma , H. Rai , D. N. S. Gautam , P. K. Prajapati , and R. Sharma , “Emerging Evidence on Omicron (B. 1.1. 529) SARS‐CoV‐2 Variant,” Journal of Medical Virology 94, no. 5 (2022): 1876–1885. 35083761 10.1002/jmv.27626 PMC9015596

[86] G. Iacobucci , “Covid‐19: Runny Nose, Headache, and Fatigue Are Commonest Symptoms of Omicron, Early Data Show,” British Medical Journal Publishing Group 375 (2021): n3103, 10.1136/bmj.n3103. 34916215

[87] R. Sarkar , M. Lo , R. Saha , et al., S Glycoprotein Diversity of the Omicron Variant (MedRxiv, 2021), https://www.medrxiv.org/.

[88] Y. Liu , J. Liu , and P.‐Y. Shi , “SARS‐CoV‐2 Variants and Vaccination,” Zoonoses 2, no. 1 (2022): 62333. 10.15212/zoonoses-2022-0001 PMC8909890 35284912

[89] M. T. Hernández‐Huerta , L. Pérez‐Campos Mayoral , C. Romero Díaz , et al., “Analysis of SARS‐CoV‐2 Mutations in Mexico, Belize, and Isolated Regions of Guatemala and its Implication in the Diagnosis,” Journal of Medical Virology 93, no. 4 (2021): 2099–2114. 33049069 10.1002/jmv.26591 PMC7675408

[90] X. Chi , R. Yan , J. Zhang , et al., “A Potent Neutralizing Human Antibody Reveals the N‐Terminal Domain of the Spike Protein of SARS‐CoV‐2 as a Site of Vulnerability,” bioRxiv (2020): [preprint].

[91] Z. Ding , T. Chen , J. Lan , and G. Wong , “Application of Animal Models to Compare and Contrast the Virulence of Current and Future Potential SARS‐CoV‐2 Variants,” Biosafety and Health 4, no. 3 (2022): 154–160. 35528630 10.1016/j.bsheal.2022.05.001 PMC9069976

[92] H. Liu , Q. Zhang , P. Wei , et al., “The Basis of a More Contagious 501Y. V1 Variant of SARS‐CoV‐2,” Cell Research 31, no. 6 (2021): 720–722. 33893398 10.1038/s41422-021-00496-8 PMC8063779

[93] J. Li , H. Jia , M. Tian , et al., “SARS‐CoV‐2 and Emerging Variants: Unmasking Structure, Function, Infection, and Immune Escape Mechanisms,” Frontiers in Cellular and Infection Microbiology 12 (2022): 869832. 35646741 10.3389/fcimb.2022.869832 PMC9134119

[94] C. Chakraborty , A. R. Sharma , M. Bhattacharya , G. Agoramoorthy , and S.‐S. Lee , “Evolution, Mode of Transmission, and Mutational Landscape of Newly Emerging SARS‐CoV‐2 Variants,” MBio 12, no. 4 (2021): e01140‐21. 34465019 10.1128/mBio.01140-21 PMC8406297

[95] Li‐Ann Wong CGY , Jahan Nowrozy Kamar , and Pillai Naganathan , “COVID‐19 Vaccine: Review of the Mechanism of Action of Different Types of Vaccine,” Open Access Library Journal 9, no. 4 (2022).

[96] B. M. Prüβ , “Variants of Sars CoV‐2: Mutations, Transmissibility, Virulence, Drug Resistance, and Antibody/Vaccine Sensitivity,” Frontiers in Bioscience (Landmark Edition) 27, no. 2 (2022): 65. 35227008 10.31083/j.fbl2702065

[97] Y. F. Hu , J. C. Hu , H. R. Gong , et al., “Computation of Antigenicity Predicts SARS‐CoV‐2 Vaccine Breakthrough Variants,” Frontiers in Immunology 13 (2022): 861050. 35401572 10.3389/fimmu.2022.861050 PMC8987580

[98] C. B. Creech , S. C. Walker , and R. J. Samuels , “SARS‐CoV‐2 Vaccines,” JAMA 325, no. 13 (2021): 1318–1320. 33635317 10.1001/jama.2021.3199

[99] N. Firouzabadi , P. Ghasemiyeh , F. Moradishooli , and S. Mohammadi‐Samani , “Update on the Effectiveness of COVID‐19 Vaccines on Different Variants of SARS‐CoV‐2,” International Immunopharmacology 117 (2023): 109968. 37012880 10.1016/j.intimp.2023.109968 PMC9977625

[100] D. D. Singh , A. Sharma , H. J. Lee , and D. K. Yadav , “SARS‐CoV‐2: Recent Variants and Clinical Efficacy of Antibody‐Based Therapy,” Frontiers in Cellular and Infection Microbiology 12 (2022): 839170. 35237535 10.3389/fcimb.2022.839170 PMC8883582

[101] W. Y. Chi , Y. D. Li , H. C. Huang , et al., “COVID‐19 Vaccine Update: Vaccine Effectiveness, SARS‐CoV‐2 Variants, Boosters, Adverse Effects, and Immune Correlates of Protection,” Journal of Biomedical Science 29, no. 1 (2022): 82. 36243868 10.1186/s12929-022-00853-8 PMC9569411

[102] K. Sharma and J. Li‐Kim‐Moy , “COVID‐19 Vaccines in 2023,” Australian Prescriber 46, no. 3 (2023): 60–63. 38053809 10.18773/austprescr.2023.020 PMC10665092

[103] E. Torbati , K. L. Krause , and J. E. Ussher , “The Immune Response to SARS‐CoV‐2 and Variants of Concern,” Viruses 13, no. 10 (2021): 1911. 34696342 10.3390/v13101911 PMC8537260

[104] B. A. S. Machado , K. V. S. Hodel , L. M. S. Fonseca , et al., “The Importance of Vaccination in the Context of the COVID‐19 Pandemic: A Brief Update Regarding the Use of Vaccines,” Vaccines 10, no. 4 (2022): 591. 35455340 10.3390/vaccines10040591 PMC9027942

